# Rapid Disease Control in First-Line Therapy-Resistant Mucous Membrane Pemphigoid and Bullous Pemphigoid with Omalizumab as Add-On Therapy: A Case Series Of 13 Patients

**DOI:** 10.3389/fimmu.2022.874108

**Published:** 2022-04-20

**Authors:** Marina Alexandre, Gérôme Bohelay, Thomas Gille, Christelle Le Roux-Villet, Isaac Soued, Florence Morin, Frédéric Caux, Sabine Grootenboer-Mignot, Catherine Prost-Squarcioni

**Affiliations:** ^1^ Department of Dermatology and Referral Center for Autoimmune Bullous Diseases (MALIBUL), Avicenne Hospital, Hôpitaux Universitaires de Paris Seine-Saint-Denis, AP-HP, Université Sorbonne Paris Nord, Bobigny, France; ^2^ Inserm UMR 1125 Li2P, UFR SMBH Léonard de Vinci, Université Sorbonne Paris Nord, Bobigny, France; ^3^ Department of Physiology & Functional Explorations, Avicenne Hospital, Hôpitaux Universitaires de Paris Seine-Saint-Denis, AP-HP, Université Sorbonne Paris Nord, Bobigny, France; ^4^ Inserm UMR 1272 “Hypoxia & the Lung”, UFR SMBH Léonard de Vinci, Université Sorbonne Paris Nord, Bobigny, France; ^5^ Department of ENT and Referral Center for Autoimmune Bullous Diseases (MALIBUL), Avicenne Hospital, Hôpitaux Universitaires de Paris Seine-Saint-Denis, AP-HP, Université Sorbonne Paris Nord, Bobigny, France; ^6^ Department of Immunology and Referral Center for Autoimmune Bullous Diseases (MALIBUL), Saint Louis Hospital, AP-HP, Université de Paris, Paris, France; ^7^ Department of Immunology and Referral Center for Autoimmune Bullous Diseases (MALIBUL), Bichat Hospital, AP-HP, Université de Paris, Paris, France; ^8^ Department of Pathology, Avicenne Hospital, Hôpitaux Universitaires de Paris Seine-Saint-Denis, AP-HP, Université Sorbonne Paris Nord, Bobigny, France; ^9^ Department of Histology, UFR SMBH Léonard de Vinci, Université Sorbonne Paris Nord, Bobigny, France

**Keywords:** omalizumab (Xolair), mucous membrane pemphigoid (MMP), bullous pemphigoid (BP), autoimmune bullous diseases (AIBDs), autoimmune skin diseases, Immunoglobulin E (IgE), Anti-BP180 IgG, Anti-BP180 IgE

## Abstract

The role of IgE autoantibodies has been demonstrated in the pathogenesis of bullous pemphigoid for many years. Recently, omalizumab (OMZ), a humanized monoclonal anti-IgE antibody that depletes total serum IgE, has been used off-label in a few case series of bullous pemphigoids demonstrating a rapid efficacy and allowing significant improvements or complete remission as add-on therapy in first-line treatment-resistant patients. Herein, we report the largest retrospective study to evaluate OMZ effectiveness in patients with subepidermal autoimmune blistering diseases. Our series included 13 patients from a single center with bullous pemphigoid or mucous membrane pemphigoid, of whom 7 had mucous membrane involvement. OMZ was added to the unchanged immunosuppressive therapies. Detailed clinical and immunological data during the first year were collected, notably for specific anti-BP180-NC16A IgE and IgG, and the median total follow-up was 30 months (range: 3–81). Our series demonstrated that OMZ induced a significant improvement in pruritus, urticarial score, and daily blister count on day 15, allowing disease control to be achieved in a 1-month median time and complete remission (CR) in a 3-month median time in 85% of these patients previously in therapeutic impasse. At the end of the follow-up, 31% of patients achieved CR on minimal therapy after OMZ weaning without relapses, and 54% achieved CR on OMZ continuation with a minimal dose of concomitant treatment. Two patients experienced therapeutic failure (15%). At baseline, clinical variables reflecting activity were significantly positively correlated with eosinophil blood count, total IgE serum level, specific anti-BP180 IgE and IgG. While baseline anti-BP180 IgG and specific anti-BP180 IgE were significantly positively correlated, only the two patients who experienced a therapeutic failure with OMZ did not fit with this correlation, demonstrating elevated levels of anti-BP180 IgG with no measurable BP180-specific IgE. Follow-up of immunological variables demonstrated a rapid decrease of eosinophilia towards normalization, whereas a slower decline towards negativation was observed over 1 year for anti-BP180 IgG and anti BP180 IgE in patients who responded to OMZ. This case series demonstrated that OMZ is a rapidly effective biologic therapy for refractory bullous pemphigoid and mucous membrane pemphigoid, permitting rapid disease control and reduction of concomitant therapeutics.

## Introduction

BP180 is the target antigen of autoantibodies in several autoimmune bullous diseases (AIBDs) including bullous pemphigoid (BP) and mucous membrane pemphigoid (MMP). In BP, the pathogenicity of anti-BP180 immunoglobulin (Ig) IgG, mast cells, and eosinophils is established for a long time (1). Currently, the pathogenicity of IgE, notably directed against the non-collagenous 16 A (NC16A) region of BP180, is also well documented in BP (2). Most BP patients have elevated circulating total IgE levels and harbor both specific anti-BP180 IgG and IgE in their sera. Circulating total and specific anti-BP180 IgE levels correlate with disease severity and clinical course. Whereas association between specific IgE and clinical phenotype remained unclear with diverging results among studies, some found specific IgE to be correlated with urticarial activity/phenotype and pruritus (2–4).

In MMP, unlike BP, the direct pathogenic role of anti-BP180 autoantibodies (IgG or IgE) has not convincingly been demonstrated. The pathogenicity of specific anti‐BP180 IgE has been suggested by studies demonstrating IgE binding to the basement membrane zone (BMZ) (5). Nevertheless, immunoblotting failed to detect specific anti-BP180 IgE in the serum of nine patients with MMP in a previous report (6).

Standard treatments for BP consist of topical or systemic corticosteroids (CS), often in combination with second-line adjuvant immunosuppressive/immunomodulatory therapies in refractory or severe cases (5). The recommended first-line treatment for MMP is dapsone, alone or in combination with topical corticosteroids, in mild/moderate cases, but combination with systemic immunosuppressants must be considered in severe cases (6). In refractory cases, adjuvant treatments might be slow to achieve disease control, leading to the long-term use of CS to reduce patient discomfort. As BP and MMP mainly affect the elderly over 60 years, contraindications to second-line therapies or adverse effects of the latter are common. Therefore, therapies with a safe profile could benefit patients with MMP or BP refractory to CS as add-on therapies in association with second-line treatments or when the latter cannot be used. Considering recent advances in the understanding of the direct pathogenic role of eosinophils and anti-BP180 and BP230 IgE, eosinophil-targeted therapies and treatment interfering with specific IgE pathogenicity are beginning to be used in BP (2).

Omalizumab (OMZ), a recombinant humanized monoclonal antibody that binds the Fc portion of IgE, has been approved for the treatment of severe asthma and chronic spontaneous urticaria with a good tolerance profile (7, 8). Through various mechanisms, OMZ reduces IgE production by B cells, decreases IgE-mediated histamine release by mast cells, basophils, and eosinophils, and reduces peripheral eosinophil count (4, 9). Since Fairley et al. described the first case of a BP patient efficiently treated with OMZ in 2009, the response to OMZ therapy in BP treatment was reported only in case reports and three case series, the largest of which involved eleven patients (10–32). Overall, OMZ successfully achieved disease control in most of these patients with BP, based on a review from 2019 (33). Nevertheless, criteria are still pending to identify patients who would benefit the most from OMZ, because variables such as treatments associated with OMZ, time to assess efficacy, definition of outcome measure, and follow-up of biological variables have been variously reported in the literature. Besides, the mechanism of action of OMZ questions its therapeutic interest in AIBDs other than BP, notably in those sharing similar clinical phenotypes or antigen targets, such as MMP in which OMZ use was not reported to date to our knowledge. We report hereinafter a case series of 13 patients with first-line therapy-resistant BP or MMP treated with OMZ, with detailed clinical, biological, and immunological follow-up over a 1-year period.

## Patients and Methods

This single-center, retrospective study was conducted on patients followed between 2014 and 2020, using the database of our referral center for AIBDs after local institutional review board approval (#CLEA-2020-140). Written informed consent for participation was not required in accordance with the French national legislation.

### Standard Assessment of AIBD in Our Reference Center

All patient data were systematically recorded using a computer medical chart standardized for AIBDs. The definitive diagnosis of AIBD and its type relied on a multidisciplinary clinical assessment recording past medical history, cutaneous and mucous membrane (MM) lesions, histological and immunological tests, as previously reported (34): direct immunofluorescence (DIF) and indirect immunofluorescence (IIF), BP180-NC16A, BP230 and collagen VII enzyme-linked immunosorbent assays (ELISAs), IgG immunoblotting performed with human amniotic membrane extract as previously described (35) and direct immunoelectron microscopy (DIEM). The multidisciplinary clinical assessment in our center included a systematic examination of ENT, oral, ocular and genital MM. In case of subepithelial AIBD (*i.e*., linear Ig or C3 deposits along the BMZ on DIF), patients were diagnosed with BP or MMP based on clinical criteria according to the criteria of Vaillant for BP (36), the predominant involvement of MM and DIEM results when the latter could be achieved (37).

### Omalizumab-Treated Patients

All patients with a diagnosis of BP or MMP who received OMZ were identified by a computer search in our database and were included in the present study.

The collegial decision to start OMZ as an off-label add-on therapy was taken considering the first reports from the literature, with the aim of achieving rapid remission of severe symptoms in patients in therapeutic impasse and/or bearing comorbidities. OMZ was added to previous treatments, of which regimen remained unchanged until disease control. OMZ dosage was calculated according to the asthma dosing nomogram, considering body weight and baseline serum total IgE level. The management of therapeutic de-escalation was the same for all cases: after reaching disease control, topical CS were progressively tapered and weaned before considering OMZ and ISA tapering, to prevent relapse and achieve complete remission (CR) on minimal therapy.

### Collected Data

Disease control (no new active lesions and beginning of healing of established lesions), CR (complete healing, and no active lesions for 2 months), and relapse were defined according to consensus statements (38, 39). Failure of therapy with OMZ was adapted from the latter and defined as no disease control after 3 months of treatment with OMZ. Baseline was defined as the date of the first OMZ injection. At baseline were collected: age, sex, type of AIBD, time between AIBD diagnosis and baseline, treatment lines before OMZ, and serum total IgE. Clinical, biological, and immunological data reflecting disease activity and adverse events were collected at baseline, at day 15, and then at 1, 2, 5, and 12 months after the first OMZ injection. At each point, were recorded: pruritus score evaluated using the pruritus visual analog scale (PVAS), bullous pemphigoid disease activity index (BPDAI), including urticaria lesions score, daily cutaneous blisters count, presence and location of MM lesions, eosinophil blood count, and anti-BP180-NC16A IgG and anti-BP180-NC16A IgE levels. The BPDAI values vary between 0 and 360 because of the addition of activity scores between 0 and 120 for each of the following components: cutaneous blisters/erosions, cutaneous urticarial/erythema lesions, and MM lesions. It was used for all patients, including those with MMP instead of the mucous membrane pemphigoid disease activity index (MMPDAI), which does not include a score of activity for cutaneous urticarial lesions.

### Total IgE and anti-BP180-NC16A Antibodies Serological Detection

Total serum IgE levels (kIU/L) were determined by ELISA (ImmunoCAP total IgE; Thermo Fisher Scientific, Whaltham, MA, USA). BP180-NC16A IgG levels were measured using a commercial ELISA kit (MBL Co., Nagoya, Japan). BP180-NC16A IgE levels were determined by an in-house ELISA. This ELISA used microplates coated with the NC16A immunocompetent domain (EUROIMMUN, Bussy-Saint-Martin, France) and was optimized (serial dilutions of the serum and the second antibody). Optimal conditions were obtained with 1:100 diluted sera and HRP-labeled anti-human IgE antibody (Southern Biotechnology, Birmingham, AL, USA) diluted 1:1000. Antibody levels were determined as mean optical density (OD) values at 450 nm after subtraction of the blank values. Subsequently, the sera of 47 BP, 32 pemphigus, and 33 healthy donors were examined, and a ROC analysis allowed the determination of the cut-off value of 5 relative units (RU)/mL with 70% sensitivity and 100% specificity, according to previous studies (40, 41).

### Literature Cases

**Figure 1:**
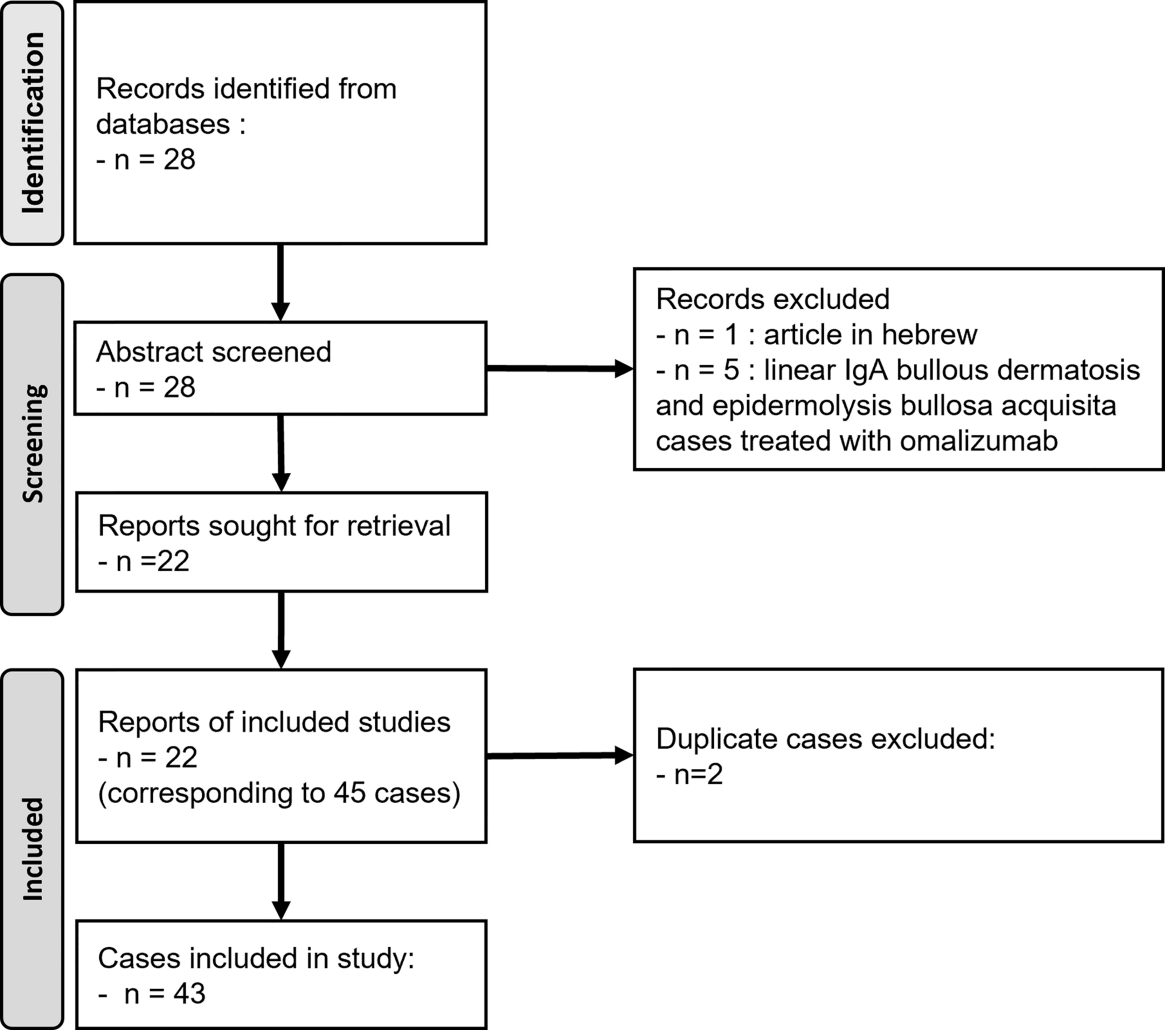
Flow diagram for the literature review on BP/MMP cases treated with omalizumab.

To better discuss our results with those obtained in previously published cases, we performed a literature review of BP and MMP cases treated with OMZ. The research was performed using PubMed and Google Scholar, using the following search terms: “omalizumab”, “bullous pemphigoid”, “mucous membrane pemphigoid”, “autoimmune bullous disease”. All reports of BP or MMP treated with OMZ, in English or French, were considered without other eligibility criteria to compare clinical and immunological data of the cases before OMZ therapy and during follow-up. The flow diagram was designed according to the PRISMA guidelines (42) (**Figure 1**).

### Statistics

Quantitative variables are presented as medians and interquartile or extreme values as indicated, or means ± standard deviation, according to normality, as assessed by the Shapiro test. Qualitative variables are presented as numbers and proportions. Statistical analyses and comparisons between baseline and day 15 were performed using the Wilcoxon matched-pairs signed rank test using Prism^®^ software (GraphPad Software Inc., San Diego, CA, USA). Correlations between quantitative variables were assessed using Spearman’s test. The Spearman correlation matrix was performed using R Statistical Software (version 2.14.0; R Foundation for Statistical Computing, Vienna, Austria).

## Results

### Study Population at Baseline

Thirteen patients were included, eight women and five men, with a mean age of 66 years (**Table 1**): 60 years old (range: 37–84) for BP patients and 76 years old (range: 57–90) for MMP patients. Eight patients had BP and five had MMP. Subepithelial AIBD diagnoses were proven by the presence of immune deposits along the BMZ in DIF in all patients but one, who was already being treated with corticosteroids when addressed in our center. Serological detection by ELISAs and immunoblotting demonstrated autoantibodies against BP180 in 12 patients (92%), BP230 in 7 patients (54%) and 120 kDa α6 integrin in 4 patients (31%). No anti-collagen VII nor laminin 332 reactivity was detected in any patient (**Table 1**).

**Table 1:** Patient characteristics at baseline.

Case No.	Sex/Age	Comorbidities	AIBD type	Disease involvement	Histological and Immunological results							AIBD duration (months)	Previous therapies	CISA	Total IgE titer (kIU/L)	Eosinophilia (cells/mm^3^))	Immunoblot IgG (kDa)
					Skin urticaria/scaring	MM	Eosinophilic skin infiltrate		Deposits along the BMZ in DIF	Deposits in DIEM	BP180 ELISA IgG/IgE (RU/mL)						
**1**	F/75	HTN, Osteoporosis	BP	Yes/No	No	important		Negative*	np	67/1	180	7	tCS, MMF	No	475	470
**2**	M/65	HTN, T2DM	BP	Yes/No	No	moderate		Linear IgG, C3	LL	187/65	180 and 120	39	tCS, CS DDS MMF	No	9,414	17,000
**3**	F/67	Sjogren’s syndrome, Cystadenolymphoma, MGUS, COPD	BP prurigo	Yes/No	No	moderate		Linear C3	np	39/41	np	14	tCS, Colchicine DOX	Yes	4,657	450
**4**	F/39	none	BP	Yes/No	No	moderate		Linear IgG, C3	np	152/200	180 and 120	2	tCS, MTX DOX	No	1,132	1,060
**5**	M/62	HTN, depression	BP	Yes/No	No	weak		Linear IgG, C3	LL	129/12	np	1	tCS, DOX	Yes	953	2,490
**6**	F/50	Asthma, Atopic dermatitis	BP	No/No	No	moderate		Linear IgG, C3	np	11/1	180	118	tCS, MTX, MMF CsA	No	878	510
**7**	M/84	T2DM, HTN, Alzheimer’s disease	BP	Yes/No	Mouth	important		Linear IgG, C3	np	201/27	180	4	tCS DOX	No	3,163	2,860
**8**	F/37	none	BP	Yes/Yes	Mouth	weak		Linear IgG, C3	LL	136/200	180 and 120	1	tCS Colchicine	Yes	964	4,790
**9**	M/61	HTN, DDS–induced psychosis	MMP	Yes/Yes	Mouth Larynx	weak		Linear IgG, C3	np	199/200	np	4	tCS DOX DDS RTX	No	4,355	1,590
**10**	F/57	none	MMP	Yes/Yes	Mouth Larynx	No		Linear IgG, C3	np	159/3	180	1	tCS DDS	No	1,749	5,350
**11**	F/90	CIHD, Breast cancer, chronic hypercalcemia chronic kidney disease	MMP	Yes/Yes	Pharynx Larynx	moderate		Linear IgG, C3	np	73/132	180	5	tCS DOX	Yes	1,308	1,840
**12**	F/86	Rheumatoid arthritis	MMP	Yes/Yes	Vulva	moderate		Linear C3	LD	18/1	180 and 120	36	tCS DOX DDS	Yes	331	230
**13**	M/85	CIHD, cerebral ischemia stroke	MMP	No/No	Mouth Larynx Genital	moderate		Linear IgG, IgA, C3	LD	1/1	np	40	CTX DDS RTX	Yes	195	200

^*^Under topical corticosteroids.

AIBD, autoimmune bullous disease; BP, bullous pemphigoid; CISA, contraindication to immunosuppressive agent; CIHD, chronic ischemic heart disease; COPD, chronic obstructive pulmonary disease; CS, systemic corticosteroids (0.5–1.0 mg/kg/d); CsA, cyclosporin; DDS, dapsone; DIEM, direct immunoelectron microscopy; CTX, cyclophosphamide; DOX, doxycycline; HTN, hypertension; ISA, immunosuppressive agent; MGUS, monoclonal gammopathy of undetermined significance; MM, mucous membrane; MMP, mucous membrane pemphigoid; MMF, mycophenolate mofetil; MTX, methotrexate; np, not acquired; OMZ, omalizumab; T2DM, type 2 diabetes mellitus; tCS, high potent topical corticosteroids (30–40 g/d).

Concurrently, total serum IgE levels were above the normal limit of 100 kIU/L in all 13 patients (100%). Eosinophil blood count was above 450 cells/mm3 in 11 of them (84%), and eosinophils infiltrating the dermis on the skin biopsy taken at diagnosis were present in 12 (92%) (**Table 1**).

Patients had mild (n = 1), moderate (n = 6), or severe (n=6) AIBDs according to BPDAI scoring (mean BPDAI: 56/360), responsible for severe suffering, evolving for a median time of 5.1 months since their diagnosis (range: 0.7–117.8) despite previous treatments; 6 patients had contraindications or had refused other immunosuppressive therapies. All patients had active skin lesions, except for case #13, who had MM involvement only. Case #3 had a prurigo variant of BP, and case #6 had a BP with severe, long-standing pruritus. Seven patients had mucosal lesions involving the buccal, laryngeal, or genital MM (**Table 1**). At baseline, 12 patients had pruritus with a median PVAS of 7 (range: 4–10), 11 had urticarial lesions with a mean urticarial score of 24.5/120 (range: 0–56), and 10 had daily blisters with a median daily blisters count of 53 (range: 0–580) (**Table 2**).

**Table 2:** Outcomes of OMZ therapy.

Case No	OMZ regimen	Concurrent therapies with OMZ	Duration of concurrent therapies before OMZ (months)	Baseline BPDAI score	PVAS		Daily blisters count		Urticarial lesions		MM lesions	Time to			Last follow–up	
					Baseline (score)	Time to normalize (days)	Baseline (number)	Time to normalize (days)	Baseline (score)	Time to normalize (days)	Time to normalize (days)	DC/CR (days)	tCS tapering/weaning(months)	OMZ tapering/weaning(months)	Time since baseline/since CR (months)	Disease control
1	450 mg Q2 wk	tCS MMF 1.5 g/d	10.3 3.1	28	6	failure	20	*na*	7	failure	*NA*	failure	No/No	no/7	55/42	CR on minimal therapy (CS)
2	600 mg Q2 wk	tCS DDS 100 mg/d MMF 3 g/d	13.4 13.4 0.8	73	8	30	400	15	41	30	*NA*	30/90	1/4	5/27	81/78	CR on minimal therapy (MMF, DDS)
3	600 mg Q2 wk	tCS	3.7	21	7	30	0	*na*	9	15	*NA*	30/90	1/3	5/no	36/33	CR on OMZ Q8 wks
4	600 mg Q2 wk	tCS DOX 200 mg/d MTX 15 mg/w	3.7 3.7 1.1	53	10	30	100	30	21	30	*NA*	30/90	1/4	15/no	23/20	CR on OMZ Q2 wks and minimal therapy (MTX)
5	300 mg Q4 wk	tCS DOX 200 mg/d	0.4 0.4	47	10	15	71	15	22	30	*NA*	30/90	1/6	15/18	33/30	CR on minimal therapy (MTX, instituted at M15 for psoriasis)
6	300 mg Q2 wk	tCS MMF 2 g/d	10.3 4.1	16	10	30	0	*na*	0	*na*	*NA*	30/90	1/4	7/no	64/61	CR on OMZ Q2 wks and minimal therapy (MMF)
7	600 mg Q2 wk	tCS DOX 200 mg/d	2.8 2.5	68	5	30	168	30	35	15	Mouth: 30	30/90	1/2	no/no	3/NA	CR on OMZ Q2 wks Death from aspiration pneumonia at M3
8	450 mg Q2 wk	tCS DOX 200 mg/d	0.8 0.8	96	5	15	53	15	56	60	Mouth: 60	60/120	1/6	3/no	10/6	CR on OMZ Q3 wks and minimal therapy (DOX)
9	600 mg Q2 wk	tCS DOX 200 mg/d RTX 2 g*	6.2 3.5 1.6	71	6	60	43	60	32	90	Mouth: 30 Larynx: 150	90/210	1/5	5/12	34/27	CR on minimal therapy (DOX)
10	600 mg Q2 wk	tCS DDS 125 mg/d RTX 2g (M0)	0.3 0.3 ¶	131	9	150	580	150	53	30	failure	failure	5/no	no/no	28/21	CR on OMZ 600 mg Q2 wks, tCS 30 g/w, DDS 50 mg/d, IVIG 2g Q3 wks, RTX (2 g at M1, M2 and M18)
11	600 mg Q2 wk	tCS DDS 100 mg/d	2.4 1.0	98	4	15	75	15	30	15	Mouth: 60 Larynx: 120	15/75	1/2	no/3	6/2	CR on DDS Death from kidney failure at M6
12	450 mg Q4 wk	tCS DDS 25 mg/d	1.6 1.2	21	7	60	5	60	3	60	Vulva: 60	60/120	*na/*2	no/no	4/NA	CR on OMZ Death from COVID–19 pneumonia at M4
13	300 mg Q4 wk	DDS 75 mg/d	32.5	5	0	*na*	0	*na*	*na*	*na*	Genital: 60 Mouth: 90 Larynx: 120	30/180	*na/na*	no/no	40/34	CR on OMZ 300 mg Q4 wks and minimal therapy (DDS)

*Previously programmed a second RTX cycle for consolidation at M4 from baseline; ¶ Rituximab was introduced 3 months after OMZ.

BPDAI, bullous pemphigoid disease area index; CR, complete remission; DC, disease control; DDS, dapsone; DOX, doxycycline; MMF, mycophenolate mofetil; M1/M12: 1 or 12 months from baseline; MTX, methotrexate; na: not acquired; OMZ, omalizumab; PVAS, pruritus visual analog scale; tCS, high potent topical corticosteroids (30–40 g/d); Q2 wks, every 2 weeks.

### Clinical Response After OMZ Adjunction and Outcome

**Figure 2:**
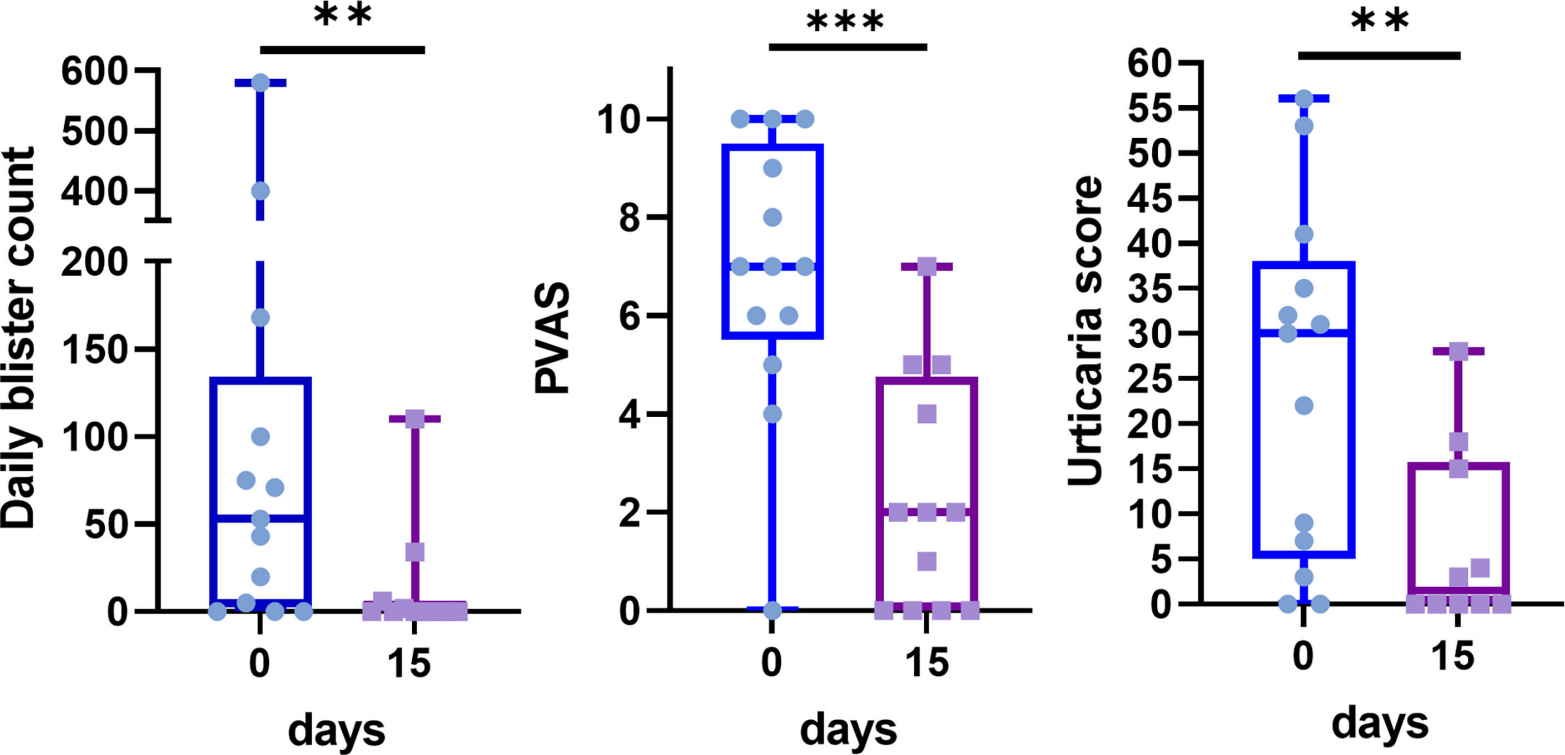
Dramatic improvement of daily blister count, pruritus visual analog score (PVAS) and urticaria score between baseline (blue) and the 15^th^ day after omalizumab therapy (purple). Data are presented as box and whisker plots showing extreme values, interquartile ranges, and medians. **p < 0.01, ***p < 0.001 (Wilcoxon signed–rank test).

**Figure 3:**
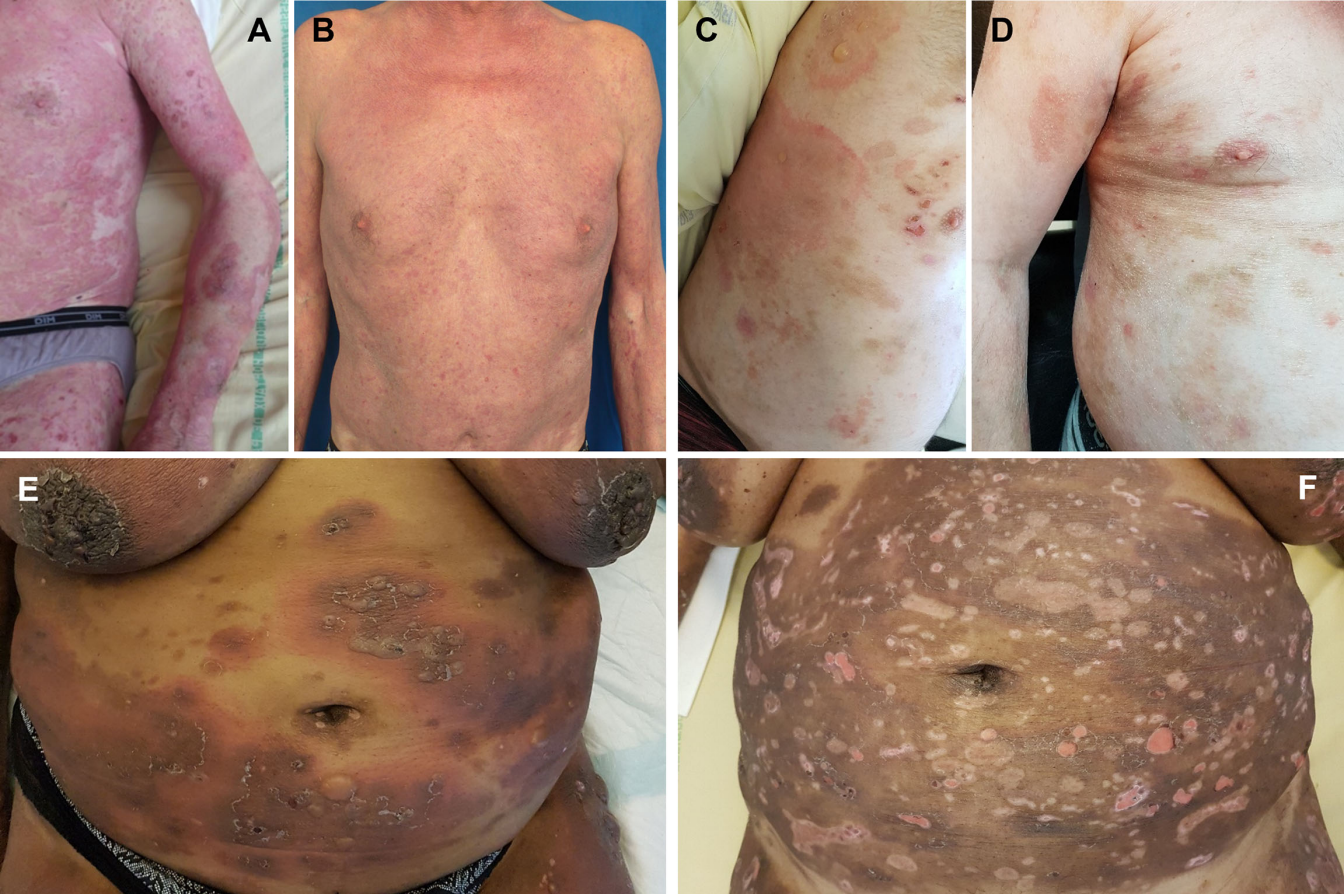
Clinical improvement during omalizumab therapy. Case #2 at baseline **(A)** and day 15 **(B)**; case #9 at baseline **(C)** and day 30 **(D)**; case #11 at baseline **(E)** and day 30 **(F)**.

Most patients experienced a rapid improvement within the first days after the initial injection, which resulted in a significant improvement from day 15 in daily blister count, PVAS, and urticarial score (**Figures 2**, **3**).

**Figure 4:**
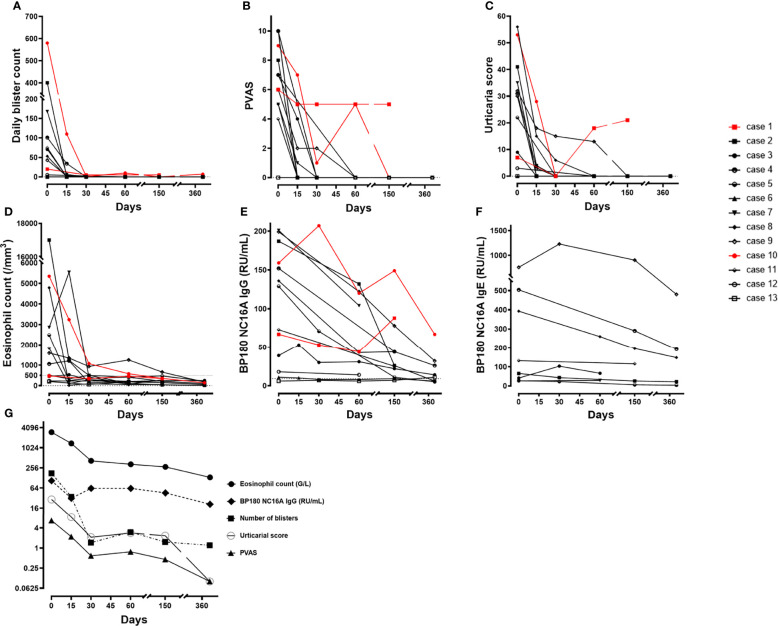
Clinical and laboratory variables during the first year after omalizumab therapy in the 13 patients. Individual values through time on a linear scale (black: remittent patients, red: non–remittent patients): **(A)** Daily blister count, **(B)** Pruritus visual analogic score, **(C)** Urticaria score, **(D)** Eosinophil count, **(E)** BP180–NC16A IgG level, **(F)** BP180–NC16A IgE level over time for the 8 patients with measurable IgE auto–antibodies during follow–up, **(G)** Log–2 scale evolution of the mean values over time of eosinophil blood count, daily blister count, pruritus visual analogic score, and BP180–NC16A IgG.

OMZ quickly achieved disease control in 11 of 13 patients (cases #2–9, #11–13) (85%) at a median time of 30 days (range: 15–90) (**Table 2** and **Figures 4A–C**), which enabled topical CS to be tapered after one month. Complete healing was obtained in these 11 patients and took longer for the 4 patients with laryngeal involvement (cases #9–11, #13) (median: 4 months) than the 3 patients (cases #7, #8, #12) with other MM lesions (median: 1.5 months) (**Table 2**). CR was achieved in 11 patients at a median time of 3 months (range: 3–7). During a mean follow-up time of 30 months (range: 3–81), none of the patients who achieved CR relapsed, whereas topical CS were weaned in a mean time of 3.8 months ± 1.6, and despite the delay between OMZ injections tapered or OMZ was weaned in 7 patients (**Table 2**). At the last follow-up, 4 of these 11 patients (cases #2, #5, #9, #11) (31%) were in CR on minimal therapy despite OMZ weaning for periods of 54, 15, 22, and 3 months, respectively (median: 18.5 months) (**Table 2**), whereas the 7 other patients (54%) were in CR on therapy with OMZ and minimal therapy dosage for other treatments for a median time of 20 months (range: 0–61).

Failure of add-on therapy with OMZ was observed in 2 patients (cases #1 and #10) (**Table 2**). OMZ did not induce any improvement and was stopped in case #1, who achieved CR with a high dosage of CS. In case 10, OMZ induced a major initial skin improvement (**Figure 2**) but was ineffective on MM (**Table 2**); however, it was continued considering initial benefit and disease severity. CR was subsequently achieved with a combination of rituximab, intravenous immunoglobulins, dapsone, OMZ, and topical CS.

OMZ therapy was administered to all patients for a mean duration of 21 months (range: 3–64). Five patients (38%) experienced adverse events. Two patients experienced mild influenza-like illness immediately after OMZ injections and were easily controlled with paracetamol, which no longer recurred after the 6^th^ injection. Three patients with comorbidities, aged between 84 and 90 years, died (cases #7, #11, and #12) from infectious pneumonia or renal failure (**Table 2**). These three patients had received OMZ for 3 to 4 months; two of them (cases #7, and #12) quickly died after having reached CR and were still on OMZ therapy at the time of their death. The third one (case #11) died at 6 months after baseline, 3 months after OMZ weaning.

### Biological Results

At baseline, the median eosinophil blood count and total IgE serum levels were 1,590 cells/mm^3^ (range: 200–17,000) and 1,132 kIU/L (range: 195–9,414), respectively. Anti-BP180 IgG level was above the normal range of 9 RU/mL in 12 patients, with a mean value of 106 RU/mL (range: 6–201). The specific anti-BP180 IgE level was above the positive cut-off value of 5 RU/mL in eight patients, with a median value of 27 RU/mL (range: 12–761). Interestingly, anti-BP180 IgE were not found in the two patients who had therapeutic failure with OMZ (cases #1 and #10) and in the three patients with the lowest levels of anti-BP180 IgG (cases #6, #12, and #13).

Biological follow-up demonstrated a rapid decrease in eosinophil blood count within the first month of OMZ therapy and returned to normal on day 150 (**Figure 4D**). After 1 year of follow-up, the mean anti-BP180 IgG titer was 23 RU/mL (range: 5–63 RU/mL). Among the 12 initially positive cases for anti-BP180 IgG, all had a progressive decrease that began within 2 months and became undetectable in 3 of them at 1-year follow-up, except for cases #1 and #10, which failed to reach disease control with OMZ and demonstrated a rebound in line with clinical activity (**Figure 4E**). Finally, the specific anti-BP 180 IgE level decreased in six of the eight positive cases at baseline and negated for one patient (case #5); cases #3 and #7 remained at the same level at the last follow-up despite a decrease in anti-BP180 IgG and CR on OMZ therapy (**Figure 4F**). Overall, these variables showed a parallel, gradually decreasing evolution towards negativation or normal levels on average (**Figure 4G**).

**Figure 5:**
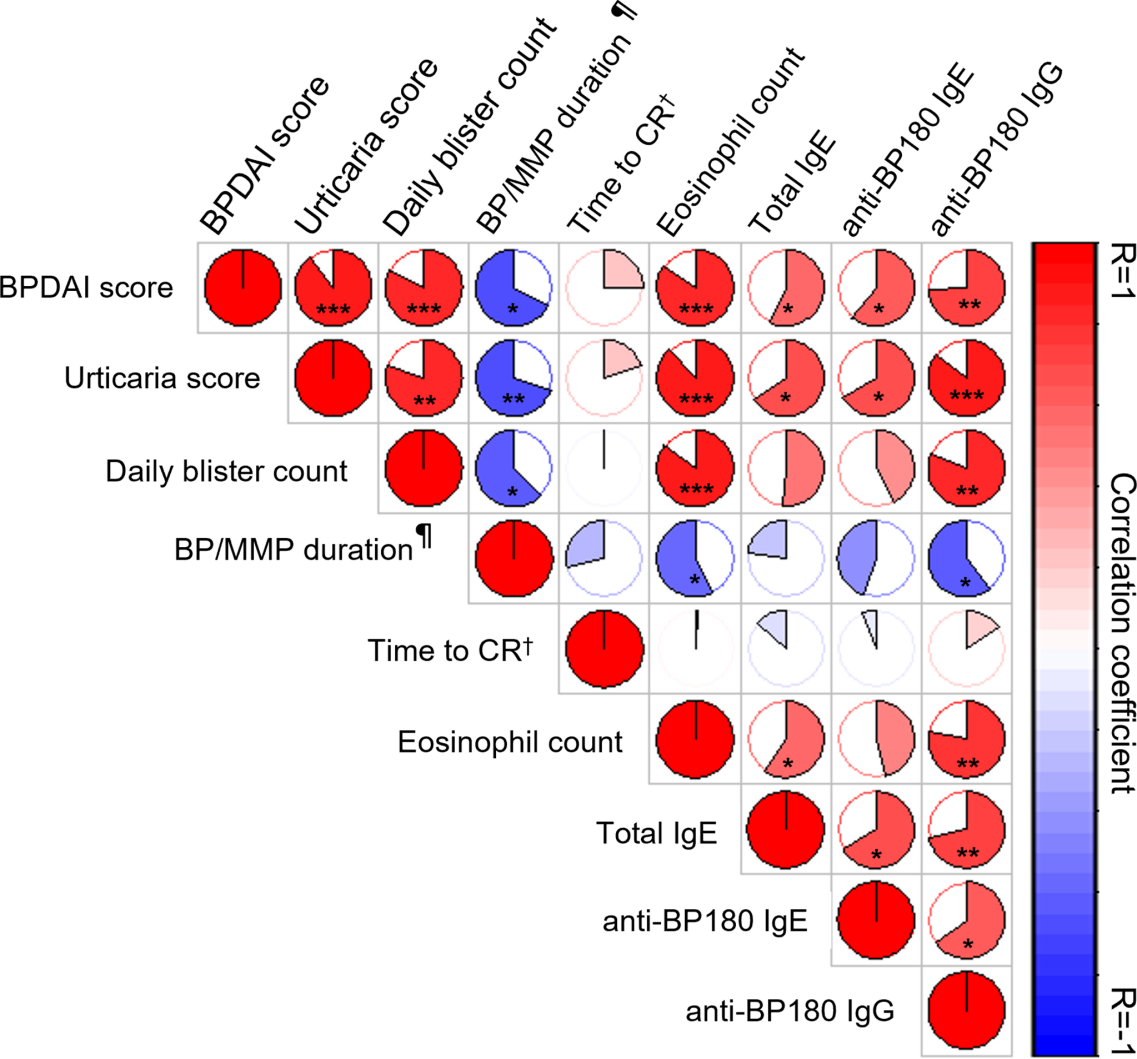
Graphical representation of Spearman correlation matrix between clinical activity and immunological variables at baseline and outcomes. Red and blue colors indicate positive and negative correlations, respectively; the lighter the color, the less significant the corresponding correlation. The filled fraction of the circle in each pie chart corresponds to the absolute value of the associated Spearman correlation coefficient (*r*). *p < 0.05, **p < 0.01, ***p < 0.001, ¶ before omalizumab, † Time to complete remission.

At baseline, clinical variables reflecting disease activity (BPDAI, urticarial score, daily blister count) were significantly positively correlated with immunological variables (eosinophil count, total serum IgE, anti-BP180 IgE and IgG) (**Figure 5**). Only the positive correlations between the daily blister count at baseline and total serum IgE or anti-BP180 IgE were not found to be significant. AIBD duration before OMZ therapy was negatively correlated with the clinical and immunological variables. No significant correlation was found between pruritus score (data not shown) or the time to achieve disease control (data not shown) or CR and other variables. Immunological variables were significantly positively correlated with each other, except for a positive correlation between eosinophil blood count and specific anti-BP180 IgE levels (**Figure 5**).

### Literature Review

The literature review identified 45 cases, of which 43 were included in our analysis (**Additional Table 1**), of which 21 were published as case series and 22 as case reports. Patients (mean age: 66.9 years ± 17.9) had treatment-resistant BP to one or several therapies, with seven cases having non-predominant MM involvement (16–18, 31). The BPDAI score was available only in a series of six patients from De et al, with a mean BPDAI score of 41 (29). Hypereosinophilia was reported in 60% of cases (median: 1,200 cells/mm^3^, range: 100–17,700). An elevated total IgE titer was observed in 74% of cases (median: 1,569 kIU/L; range: 73–22,682 kIU/L). Subcutaneous OMZ was administered at a dose of 300 mg (81%), with an initial injection interval of 2–6 weeks. Most patients (86%) received OMZ in combination with concomitant immunosuppressive therapies, including systemic CS (79%), high potent topical CS (35%), and azathioprine (14%).

Short-term results within the first month were available in only 16 patients (37%), of whom 87% demonstrated a quick improvement and achieved partial improvement (50%) or disease control (37%). Middle-term results, between the second and the fourth month after OMZ, demonstrated that 26 patients achieved disease control (60%), with CR on OMZ (53%), or CR off after a single OMZ injection in 2 patients. The specific outcomes of MM lesions have not been described. Adverse events were noted in 12% of cases; however, their direct relationship with OMZ therapy was not clear.

At the last follow-up, CR was not achieved in 11 cases (25%), with only partial improvement (16%) or therapeutic failure (7%). The best outcome achieved during follow-up was a CR in 32 cases (74%), with a 6-month median time for CR (range: 2–42 months). Disease control allowed concomitant therapies to be tapered or weaned in most of these patients. OMZ was discontinued in seven patients, resulting in a 71% relapse rate, whereas only one relapse was reported on OMZ continuation. Immunological follow-up data were sparse in most cases, showing normalization of eosinophilia and a decrease in total IgE and anti-BP180 IgG. Quantitative (ELISA) or qualitative (immunoblot) measurement of specific anti-BP180 IgE was carried out in only 2 and 7 cases, respectively (10, 11, 29) and were positive in 4 patients, of whom one had negativation at 3-month follow-up (29).

## Discussion

AIBD management can be difficult, especially in treatment-resistant cases requiring rapid relief of pruritus, blisters, and erosions. The role of IgE autoantibodies has been demonstrated in BP in recent years, which led to the off-label use of OMZ, a humanized monoclonal anti-IgE antibody depleting total serum IgE, in refractory BP patients. Here, we report the largest retrospective series of AIBDs treated with OMZ, involving 13 patients with precise short- and long-term clinical and immunological outcomes, and compare these results with the data of 43 BP cases in the literature. Our study has obvious limitations, including a small sample size and monocentric recruitment. Nevertheless, our case series with detailed follow-up and homogenous therapeutic management strengthens the previous data on OMZ efficacy in first-line therapy-resistant BP and highlights the efficacy of OMZ in MM lesions, notably in MMP patients, which was not previously reported.

### Clinical and Immunological Data at Baseline

Our series included 13 first-line treatment-refractory patients, 8 with BP and 5 with MMP, with a 66-year-old mean age. The subgroup of patients with BP was young (mean age: 60 years) in comparison with the mean age of BP patients older than 80 years in a large French cohort (43). Overall, our patients had moderate to severe disease according to the cut-off values of the BPDAI score defining the severity categories in BP (44), with a higher median BPDAI score than the cohort used to elaborate these cut-off values (53 and 37.5, respectively). In addition, our patients experienced severe pruritus with a mean PVAS of 7/10, whereas a recent series investigating pruritus and quality of life in BP described a mean pruritus intensity of 5.2/10 and showed that patients with a score above 5 had a greater alteration in their quality of life (45). As expected, our series included patients positive for anti-BP180 IgG (92%). IgE levels and eosinophil blood count were not decision criteria as such to start OMZ. Nevertheless, 100% of our patients had elevated total serum IgE, and 84% had elevated eosinophil blood count. We found elevated specific anti-BP180 IgE in eight patients, including six out of eight BP patients (75%) and in two out of five patients with MMP (40%).

In comparison with the previously published BP cases treated with OMZ, our series included patients of similar age, with a higher proportion of patients with MM involvement considering the inclusion of patients with MMP, notably 3 with laryngeal involvement. Most literature cases lacked BPDAI score and pruritus severity evaluation. Nevertheless, the mean BPDAI score in our series was similar to that reported by De et al., with a mean BPDAI score higher than 50 (29). The rates of cases with elevated total serum IgE and eosinophil blood count were slightly higher in our study. With our in-house anti-BP180 IgE ELISA, we were the first to demonstrate specific anti-BP180 IgE levels in MMP patients at baseline. The rate of anti-BP180 IgE in BP patients (75%) found in our series with an in-house ELISA was higher than in the study of De et al. who reported anti-BP180 IgE in 2 of 6 patients’ sera tested with an immunoblotting technique (29). A decrease in anti-BP180 IgE after OMZ therapy was reported in 6 out of 8 patients in our study, whereas an increase in optical density at 3 months in one patient has been reported in the only quantitative assessment after OMZ available in the literature (19). The rate of anti-BP180 IgE negativation at last follow-up was low (1 out of 8 patients) in our series, whereas only one patient negated immunoblot reactivity from the 2 positive ones at baseline in the series from De et al. (29).

Similar to most studies investigating this issue in BP (2), we found significant positive correlations between disease severity in BP/MMP and immunological variables at baseline (circulating eosinophils, total serum IgE levels, anti-BP180 IgE and IgG levels). Circulating anti-BP180 IgG was significantly correlated with urticaria and blistering. While specific anti-BP-180 IgE was inconsistently associated with clinical phenotype (e.g., urticarial or nodular) in previous studies (2), specific anti-BP180 IgE levels were significantly correlated only with the urticaria score in our series. Moreover, we found that anti-BP180 IgG and specific IgE levels were positively correlated at baseline. Interestingly, only two patients with elevated levels of anti-BP180 IgG but absence of anti-BP180 IgE were both OMZ therapeutic failures (see below).

### Clinical Response to Omalizumab

In our series, OMZ significantly reduced clinical symptoms (pruritus, urticaria, and blisters) from day 15, and disease control was achieved in a median time of 30 days. Furthermore, we reported for the first time OMZ efficacy on MM involvement, with six patients (86%) having experienced complete re-epithelialization. The latter was obtained in a shorter time for buccal and vulvar involvement than for laryngeal lesions. At the last follow-up, an 85% CR rate and a 15% therapeutic failure rate were observed. The 3-month median time to achieve CR was short, considering that these patients were resistant to previous treatments and that 7 cases had MM involvement. Indeed, two BP cases in our series had mucosal lesions that responded more slowly to conventional therapy (46). In addition, five patients had MMP, whereas the time to obtain CR with intravenous immunoglobulins or rituximab in MMP without ocular involvement was estimated at 22 and 5 months, respectively, in a recent review. Complete resolution was obtained in 77.8% and 50.0% of patients, respectively, with intravenous immunoglobulins and rituximab (47). Moreover, as naso-pharyngo-laryngeal endoscopy is an invasive procedure, it was not repeated before day 60, then day 120; thus, the time to achieve CR might have been overestimated in our three patients with laryngeal involvement. Lastly, 54% of patients achieved CR on minimal therapy with OMZ continuation and 31% CR on minimal therapy after OMZ weaning without any relapse, with a median follow-up time of 18.5 months in these latter.

Overall, our results reinforce the evidence for the rapid effectiveness of OMZ, as already outlined in the literature. Our series specified information highlighting its rapid improvement through systematic monitoring; notably the striking decrease of clinical symptoms at day-15, a one-month median time to achieve disease control and a 3-month median time to reach complete remission. At the last follow-up, an 85% CR rate and a 15% therapeutic failure rate were observed, which were slightly better than the outcomes from the literature review we performed, which demonstrated 74% and 25% rates, respectively. A third of our patients achieved CR on minimal therapy after OMZ weaning, without relapse, while a high relapse rate after OMZ withdrawal was reported in the literature. This difference might be explained by a slower decrease in therapies that only began after disease control. Notably, topical CS was slowly tapered over 4 months, according to the French guidelines for the therapeutic management of BP (48). OMZ tapering was initiated only after CS weaning in all patients with CR. Indeed, as in other indications, such as urticaria, the modalities of tapering and weaning for OMZ need to be clarified. In fact, as in the literature, a majority of patients still continued OMZ at the last follow-up for long periods, which allowed concomitant therapies cessation or tapering to their minimal dosage to prevent relapse. This continuation for long periods might represent the greatest barrier to the widespread use of OMZ considering its cost.

The target population for OMZ in patients with AIBD would also need to be clarified, but as our group size was small, with a large majority of patients responsive to OMZ therapy, we could not ascertain predictive factors of response to OMZ. No variable tested in the correlation matrix reached statistical significance with the time to achieve disease control or complete remission. Thus, as previously described by Lonowski et al. (23), we cannot conclude that patient selection for OMZ therapy should hinge upon the level of urticaria lesions, eosinophil count, or total serum IgE. Nevertheless, only two patients with very high levels of anti-BP180 IgG but no anti-BP180 IgE had therapeutic failure with OMZ. Thus, this immunological combination might constitute a negative predictive factor for the response to OMZ. This association has not previously been described in the literature but only a very few studies (10, 11, 19, 29) investigated anti-BP180 IgE, and most of them performed immunoblot analysis rather that anti-BP180 IgE ELISA. Lastly, only one case reported by Dufour et al. demonstrated elevated anti-BP180 IgG titers in ELISA without IgE in immunoblot (11), and one case from the series of De et al. showed anti-BP180 IgG positivity and anti-BP180 IgE negativity in immunoblotting (29). In these cases, OMZ allowed a significant improvement or a complete response.

Our series is the first to report the results of OMZ therapy in MM involvement, especially in patients with MMP diagnoses confirmed on DIEM. OMZ was found to be rapidly effective to decrease skin symptoms in all MMP patients who had skin involvement at baseline. OMZ as add-on therapy allowed a CR in four of the five MMP patients, notably in patient #13 having only MM involvement with normal eosinophil blood count and a moderate elevation of total IgE. After a striking improvement, patient #10 was classified as a failure because the disease relapsed and required additional therapies with OMZ continuation.

### Omalizumab Tolerance - Adverse Events

The good tolerance profile of OMZ is well documented in asthma and chronic urticaria, even in patients older than 65 years (49, 50), notably in comparison with other immunosuppressive therapies (42). Adverse events were observed in 5 patients (38%) in our series, which was higher than the adverse event rate reported in the literature (12%). This difference was not related to a longer follow-up duration, as all adverse events occurred within the first 6 months of OMZ therapy. Two patients experienced grade 2 drug-related adverse events (influenza illness reactions). Three grade 5 adverse events (two pneumonias and one renal failure) led to death in three very fragile and old patients with significant comorbidities, without apparent direct relationship between OMZ therapy and death, even more as OMZ was stopped 5 months before death in one of these three patients.

### Immunological Monitoring After Omalizumab

For the first time, we provided the time-course of immunological variables in AIBD patients under OMZ throughout the follow up over one year. As previously described in BP (51), the eosinophil blood count and anti-BP180 IgG level seemed to follow the course of clinical variables. Notably, the eosinophil blood count showed a dramatic decrease within the first month, mirroring the rapid decrease in urticaria and pruritus. The decrease in anti-BP180 IgG level was slower and progressive over the one-year follow-up for BP patients but also for MMP patients positive at baseline; most of them still demonstrated positive levels of anti-BP-180 IgG at one-year follow-up which was already described in BP patients with other therapies such as B-cell depletion therapy with rituximab (52). A similar slow decrease was observed for anti-BP180 IgE in patients with high anti-BP180 IgE levels at baseline, whereas other patients in CR had stable levels despite OMZ. As previously hypothesized for anti-BP180 IgG (53), our results suggest the need for maintenance therapy to prevent relapses in the absence of rapid or complete clearance of anti-BP180 IgE under OMZ. This could also explain the high rates of patients still on OMZ or off OMZ with minimal dosage of concomitant therapies at the end of follow-up in our series.

In conclusion, add-on therapy with OMZ allowed BP patients and MMP patients in situations of therapeutic impasse to pass a milestone, achieving fast improvement within the first 2 weeks, disease control at 1 month and a 85% rate of CR. Our results and those from the literature should be confirmed by larger retrospective and controlled studies, which might more accurately precise OMZ efficacy in late observation endpoints, such as CR on minimal therapy, and better identify the predictive factors of therapeutic response. Considering predictive factors, our results point to the need to pay particular attention to patients with a dissociation between a high anti-BP180-NC16A IgG level and the absence of specific anti-BP180-NC16A IgE levels. Although OMZ was found to be safe in large studies for other indications, future studies should confirm this good safety profile in the elderly, as we reported apparently non-OMZ-related deaths in three old patients. Finally, in patients with refractory BP and MMP, OMZ is an effective option to control their diseases and to stop or decrease concomitant immunosuppressive therapies to their minimal effective dose.

## Data Availability Statement

The raw data supporting the conclusions of this article will be made available by the authors, without undue reservation.

## Ethics Statement

The studies involving human participants were reviewed and approved by Comité Local d’Ethique pour la Recherche Clinique des HUPSSD Avicenne-Jean Verdier-René Muret (CLEA #2020-140). Written informed consent for participation was not required for this study in accordance with the national legislation and the institutional requirements. Written informed consent was obtained from the individual(s) for the publication of any potentially identifiable images or data included in this article.

## Author Contributions

MA, GB, and CPS contributed to the conception and design of the study. MA, GB, CL, IS, FC, and CPS participated in patients’ daily care and data collection. FM and SGM performed the immunological and biological analyses. MA and GB reviewed the charts and organized the databases. GB and TG performed statistical analyses and figure preparation. MA wrote the first draft of this manuscript. GB and SGM wrote sections of the manuscript. All authors contributed to manuscript revision, read, and approved the submitted version.

## Conflict of Interest

The authors declare that the research was conducted in the absence of any commercial or financial relationships that could be construed as a potential conflict of interest.

## Publisher’s Note

All claims expressed in this article are solely those of the authors and do not necessarily represent those of their affiliated organizations, or those of the publisher, the editors and the reviewers. Any product that may be evaluated in this article, or claim that may be made by its manufacturer, is not guaranteed or endorsed by the publisher.

## Abbreviations

AIBD, auto-immune blistering disease; BMZ, basement membrane zone ; BP, bullous pemphigoid ; BP180, bullous pemphigoid 180 kDa antigen; BP180-NC16A, non-collagenous 16 A region of BP180; BPDAI, bullous pemphigoid disease activity index; CS, corticosteroids; DIEM, direct immunoelectron microscopy; ELISA, enzyme-linked immunosorbent assay; Ig, immunoglobulin; MM, mucous membrane; MMP, mucous membrane pemphigoid; OMZ, omalizumab; PVAS, pruritus visual analog scale.
