# Supplementary material for: Rapid Disease Control in First-Line Therapy-Resistant Mucous Membrane Pemphigoid and Bullous Pemphigoid with Omalizumab as Add-On Therapy: A Case Series Of 13 Patients
Source: Front Immunol. 2022 Apr 20;13:874108. doi: 10.3389/fimmu.2022.874108 (PMC9065717; doi:10.3389/fimmu.2022.874108)
Supplement: Supplementary file 1 [file Table_1.docx]

**Additional Table 1 | Clinical, immunological and outcome data available for the refractory BP patients treated by OMZ in the literature.**

Abbreviations: AZA: azathioprine; AZM: azithromycin; BP: bullous pemphigoid; CR: complete remission; CR on: complete remission with OMZ continuation; CR off: CR without OMZ continuation; CS: oral or intravenous corticosteroids; DC on: disease control with OMZ; DDS: dapsone; DOX: doxycycline; F: female; IVIG: intravenous immunoglobulins; M: male; MMF: mycophenolate mofetil; ND: no data; OMZ: omalizumab; PI: partial improvement; Q2 wks: every 2 weeks; RTX: rituximab; tCS: topical corticosteroids.

| **Case Number / First author, year** | **Clinical and biological data at baseline** | | | | | | **1st**  **month**  **outcome** | **2-4**  **months**  **outcome** | **Last outcome** | | | | | **Biological Follow up** | **Side effects** |
| --- | --- | --- | --- | --- | --- | --- | --- | --- | --- | --- | --- | --- | --- | --- | --- |
|  | **Age/Sex** | **BP duration (months)** | **Eosinophils blood count (G/L or %)** | **Total IgE (IU/mL)** | **OMZ regimen** | **Add-on therapies** |  |  | **Concomitant therapies** | **OMZ regimen**  **tapering** | **Relapse**  **after**  **tapering** | **Best outcome**  **during follow-up** | **Best outcome duration (months)** |  |  |
| #1/Fairley, 2009 | 70/F | 12 | 3.42 | 222 | 300mg, Q2 wks | no | PI | PI | No | weaning  (6 months) | Yes | CR on OMZ  Relapse : AZA and CS reinstituted | 5 | Eosinophilia: normalized  Anti-BP180 IgG: decreased | - |
| #2/Dufour, 2012 | 0.5/ND | 0.5 | 13 | 636 | 100 mg, Q2 wks | CS  AZM DDS | DC on | CR on | CS  tapering | no | ND | CR on OMZ | 7 | Eosinophilia: normalized  Anti-BP180 IgG: decreased | - |
| #3 /  London, 2012 | 76/F | 6 | 1.64 | 287 | 300 mg, Q4 wks | CS  AZA | DC on | CR on | CS and AZA tapering | no | ND | CR on OMZ  Relapse: CS reinstituted and MMF | 42 | ND | - |
| #4 /  Yalcin, 2014 | 28/M | ND | ND | 5000 | 300mg, ND | ND | ND | PI | ND | no | ND | CR on OMZ | 10 | ND | - |
| #5-8 /  Yu, 2014 | 78/F | 18 | 0.12 | 1835 | 300 mg, Q6 wks | CS  AZA  DOX | ND | CR on | CS and AZA weaning | weaning  (3 months) | Yes | CR on OMZ Q4 wks  after OMZ reinstitution | 16 | ND | - |
|  | 72/F | 42 | 5.4 | 1181 | 375 mg, Q4 wks | CS | ND | CR on | CS tapering | no | ND | CR on OMZ | 9 | ND | - |
|  | 86/F | ND | 1.81 | 2135 | 375 mg, Q2 wks | no | PI | ND | No | single dose | ND | PI | 1 | ND | - |
|  | 55/F | 7 | 17.7 | 5821 | 375 mg, Q2 wks | CS  AZA | ND | CR on | CS and AZA tapering | weaning  (3 months) | Yes | CR on OMZ  Relapse : CS increased, AZA reinstituted | 3 | Eosinophilia: normalized | - |
| #9-10/ Balakirski, 2016 | 40/F | 12 | 5.50% | 1697 | 300 mg, Q4 wks | CS | PI | PI | CS  tapering | no | No | PI | 15 | Eosinophilia: stable Total IgE titer: decreased  Anti-BP180 IgG: increased | - |
|  | 63/F | > 6 | 1.10% | 1074 | 300 mg, Q3 wks | CS | PI | PI | CS weaning | weaning | Yes | PI on OMZ Q3 wks | 9 | Eosinophilia: increased  Total IgE: increased  Anti-BP180 IgG: decreased | - |
| #11 /  Gönül, 2016 | 70/F | 6 | 2.90% | 2500 | 300 mg, Q4 wks | CS | DC | CR on | CS  tapering | weaning  (7 months) | Yes | CR on OMZ Q4 wks | 8 | Total IgE: decreased | - |
| #12 /  Biglic, 2017 | 25/M | 36 | high | 1598 | 525 mg, Q2 wks | CS | PI | failure | RTX and CS tapering | weaning  (4 months) | - | failure | - | Eosinophilia: stable Total IgE: decreased | - |
| #13-23 / Incel Uysal, 2017 | 77/F | 48 | 0.1 | 4 on 11 above normal (no details for each case) | 300 mg, Q4 wks | CS  AZA | ND | ND | CS and AZA  tapering | tapering | No | CR on OMZ Q5 wks | 12 | ND | - |
|  | 81/F | 14 | 0.7 |  | 300 mg, Q2 wks | CS  AZA | ND | ND | CS tapering  AZA weaning | no | - | CR on OMZ Q3 wks | 15 | ND | - |
|  | 74/M | 2 | 1 |  | 300 mg, Q2 wks | tCS | ND | ND | tCS weaning | tapering | No | CR on OMZ Q4 wks | 4 | ND | - |
|  | 86/M | 9 | 1 |  | 300 mg, Q2 wks | CS | ND | CR on | CS weaning | tapering | No | CR on OMZ Q8 wks | > 6 | ND | - |
|  | 84/F | 1 | 1.4 |  | 300 mg, Q4 wks | CS | failure | ND | CS | single dose | - | failure | - | ND | Elevated liver enzymes |
|  | 79/M | 1 | 1.4 |  | 300 mg, Q4 wks | tCS | failure | ND | tCS | single dose | - | failure | - | ND | Myocardial infraction |
|  | 83/M | 12 | 0.1 |  | 300 mg, Q2 wks | CS | ND | PI | CS | No | - | PI | 3 | ND | Thrombo-cytopenia |
|  | 86/F | 1 | 3.8 |  | 300 mg, Q2 wks | tCS | ND | CR on | tCS tapering | tapering | No | CR on OMZ Q4 wks | 5 | ND | - |
|  | 58/M | 1 | 0.1 |  | 300 mg, Q2 wks | tCS | ND | CR on | tCS tapering | tapering | No | CR on OMZ Q4 wks | 9 | ND | - |
|  | 81/M | 18 | 0.9 |  | 300 mg, Q2 wks | CS | ND | CR on | CS tapering | tapering | No | CR on OMZ Q4 wks | 9 | ND | - |
|  | 77/F | 36 | 0.2 |  | 300 mg, Q4 wks | CS | ND | ND | CS | ND | ND | ND | ND | ND | - |
| #24 / Menzinger, 2018 | 76/F | ND | 1.45 | 4994 | 300 mg, Q4 wks | tCS | PI | DC | tCS weaning | weaning  (7 months) | Yes | CR on OMZ Q4 | 5 | Eosinophilia: normalized  Total IgE: stable  Anti-BP180 IgG: decreased | - |
| #25 /  James, 2018 | 72/M | 5 | 2.19 | 6241 | 300 mg, Q3 wks | RTX  CS  tCS | DC | CR on | RTX, tCS and CS weaning | no | - | CR on OMZ Q3 | 9 | Eosinophilia: normalized  Anti-BP180 IgG: stable | - |
| #26 /  Vico-Alonzo, 2019 | 81/M | 5 | normal | 235 | 300 mg, Q4 wks | CS  AZA  tCS | ND | ND | CS and AZA weaning | tapering | - | CR on OMZ Q7 | > 6 | Total IgE titer: decreased | - |
| #27 /  Maglie, 2019 | 60/M | 24 | ND | ND | 300 mg, Q3 wks | CS  RTX  IVIG | ND | DC | RTX, CS minimal therapy | weaning  (4 months) | No | CR off OMZ  (at 5 months) | 3 | ND | - |
| #28-29 / Seyed, 2019 | 60-65/ND | ND | 0.62 | 1176 | 300 mg, Q2 wks | tCS | ND | CR on | tCS weaning | no | - | CR on OMZ Q2 wks | ND | Eosinophilia:  normalized  Total IgE: increased  Anti-BP180 IgG: decreased | - |
|  | 70-75/ND | ND | 0.34 | 410 | 300 mg, Q4 wks | tCS | ND | CR on | tCS weaning | no | - | CR on OMZ Q4 wks | ND | Eosinophilia: increased Total IgE: increased  Anti-BP180 IgG: stable | - |
| #30 /  Ewy, 2019 | 74/F | ND | 47.30% | ND | 300mg, Q4 wks | tCS  DOX | ND | PI | ND | ND | - | PI | 17 | ND | - |
| #31 /  Sinha, 2020 | 44/F | 3 | 5.5 | 11579 | 450 mg, single dose | CS  AZA | DC | CR off | CS and AZA tapering | single dose | No | CR off OMZ | 9 | Eosinophilia: normalized  Total IgE: decreased | - |
| #32 /  Navarro Trivino, 2020 | 70/M | 3 | 0.59 | 7027 | 300 mg, Q3 wks | ND | ND | CR on | ND | weaning  (6 months) | No | CR off OMZ | 6 | ND | - |
| #33 /  Garido, 2020 | 76/F | 2 | 0.63 | 265 | 300 mg, Q4 wks | ND | DC | CR on | ND | tapering | No | CR on OMZ Q6 wks | 6 | Eosinophilia normalized Total IgE: normalized Anti-BP180 IgG: decreased | - |
| #34-35 / Mangin, 2021 | 80/F | ND | ND | ND | 600 mg, ND | MMF | PI | CR off | MMF | single dose | - | PI | ND | ND | Acquired hemophilia |
|  | 64/M | ND | ND | ND | 600, ND | CS | PI | ND | CS | single dose | - | PI | ND | ND | Acquired hemophilia |
| #36 /  Sarrazin, 2021 | 68/M | >12 | 2.53 | 1540 | 300 mg, Q4 wks | tCS  DDS  RTX | ND | DC | RTX and tCS weaning  DDS continued | No | - | CR on OMZ Q4 wks | ND | ND | - |
| #37-42 /  De, 2021 | 62/F | 3 | ND | 1151 | 300 mg, Q4 wks | tCS | ND | CR on | tCS | No | - | CR on OMZ Q4 wks | 2 | Anti-BP180 IgG: stable | - |
|  | 85/F | 2.5 | ND | 409 | 300 mg, Q4 wks | CS | ND | CR on | CS | No | - | CR on OMZ Q4 wks | 2 | Anti-BP180 IgG: stable | - |
|  | 59/M | 9 | ND | 22682 | 300 mg, Q4 wks | tCS  CS | ND | CR on | tCS, CS | No | - | CR on OMZ Q4 wks | 2 | Anti-BP180 IgG: stable | - |
|  | 58/F | 5 | ND | 21630 | 300 mg, Q4 wks | Tcs | ND | CR on | tCS | No | - | CR on OMZ Q4 wks | 2 | Anti-BP180 IgG: negativation | - |
|  | 62/F | 2.5 | ND | 4217 | 300 mg, Q4 wks | No | ND | CR on | No | No | - | CR on OMZ Q4 wks | 2 | Anti-BP180 IgG: negativation | - |
|  | 61/M | 6 | ND | 133 | 300 mg, Q4 wks | CS | ND | CR on | CS | No | - | CR on OMZ Q4 wks | 2 | Anti-BP180 IgG: stable | - |
| #43 /  Seyed, 2021 | 70/M | 36 | normal | 73 | 300 mg, Q4 wks | MMF  tCS | ND | PI | MMF and tCS weaning Dupilumab continued | No | - | CR on  OMZ and dupilumab | 3 | Anti-BP180 IgG: decreased | - |
